# Global survey on point-of-care ultrasound (pocus) use in child surgery

**DOI:** 10.1007/s00383-024-05797-8

**Published:** 2024-09-05

**Authors:** Gerlin Naidoo, Mohammed Salim, Andrew Jackson, Ashok Handa, Kokila Lakhoo, Judith Lindert

**Affiliations:** 1 Nuffield Department of Surgical Sciences, University of Oxford, John Radcliffe Hospital, Room 6607, Level 6, Headington, Oxford, OX3 9DU UK; 2 Paediatric Surgery Unit, Muhimbili National Hospital, Dar es Salaam, Tanzania; 3 Muhimbili University of Health and Allied Sciences, Dar es Salaam, Tanzania; 4 Department of Pediatric Surgery, University of Rostock, Rostock, Germany

**Keywords:** POCUS, Point-of-care ultrasound, Pediatric surgery, Bedside ultrasound, Clinical ultrasound

## Abstract

**Purpose:**

To undertake a global assessment of existing ultrasound practices, barriers to access, point-of-care ultrasound (POCUS) training pathways, and the perceived clinical utility of POCUS in Child Surgery.

**Methods:**

An electronic survey was disseminated via the GICS (Global Initiative of Children’s Surgery) network. 247 anonymized responses from 48 countries were collated. 71.3% (176/247) worked in child surgery.

**Results:**

Ultrasound was critical to practice with 84% (147/176) of requesting one daily or multiple times per week. Only 10% (17/176) could access emergency ultrasound < 1 h from request. The main barrier was a lack of trained personnel. HIC surgeons were more likely to have ultrasound training (24/29; 82.8%) compared with LMICs (74/147; 50.3%) (p = .001319; CI 95%). Self-perceived POCUS competence was associated with regularity of POCUS use (*p* < 0.001; CI 95%). Those who already practice POCUS most commonly use it for trauma, intussusception, and ultrasound-guided procedures. Majority (90%; 159/176) of child surgeons would attend formal POCUS training if available.

**Conclusions:**

Ultrasound is critically important in children’s surgery globally, however, many surgeons experience barriers to timely access. There is a strong interest in learning POCUS for relevant pediatric surgical applications. Further research is needed to evaluate the best methods of training, accreditation, and governance.

**Supplementary Information:**

The online version contains supplementary material available at 10.1007/s00383-024-05797-8.

## Introduction

Point-of-care ultrasound (POCUS) or ‘clinician-led’ ultrasound refers to the use of ultrasound by non-radiologist clinicians at the patient’s bedside. Although for many years ultrasound has been the domain of radiologists and specialist sonographers, several critical developments have occurred that have altered the clinical landscape. Technological advances have facilitated the development of cheaper and increasingly smaller handheld devices that interface wirelessly with most screens [1]. The user-friendliness of ultrasound devices in part has contributed to the rapid expansion of POCUS into almost all areas of clinical medicine [2–7]. The specialists who have led the way include Emergency Physicians and Intensivists who have developed POCUS curricula, courses, and accredited training programs for their respective fields [8–10]. The value of POCUS to Anesthesia through facilitating vascular access, cardiovascular monitoring, and guiding regional anesthesia has also been demonstrated [8, 11]. Similarly, acute gynecological presentations and obstetric care have utilized POCUS in their diagnostic algorithms and have a large body of evidence to support its use [5, 12].

The field of Pediatric Surgery could stand to benefit from this technology, given the large proportion of pathologies amenable to ultrasound diagnosis, the conducive body habitus of children, and the importance of avoiding exposure to ionizing radiation. In emergency medicine in particular, many studies have shown equivalent diagnostic accuracy when emergency physicians have been trained to diagnose appendicitis, intussusception, and pyloric stenosis [13–15]. While there is evidence of some pediatric surgeons practicing POCUS in Europe [6], and sporadic reports from other regions [14, 16–19]; overall there is a paucity of published data regarding current POCUS practices in pediatric surgery worldwide.

While the majority of POCUS published literature exists in high-income countries (HICs), theoretically low-resourced healthcare settings also stand to benefit from the ‘task shifting’ and ‘capacity-building’ advantages POCUS offers. A particular challenge in LMICs is a critical lack of radiologists. Of 26 HICs evaluated, the average number of radiologists is 100 per million population [20–24]. Compared with studies evaluating countries in Africa, where the estimated number of radiologists is between 6.5 and 12 per million [20–24]. The practice of pediatric radiology is especially neglected, with only 4 of 54 countries in Africa offering sub-specialization in pediatric radiology (Ethiopia, Nigeria, South Africa, Tunisia) [25].

Using just one example, intussusception is a common emergency pediatric surgical condition for which there is a significant disparity in outcomes between high and low-income countries [26]. In LMICs, the mortality rate is greater than 10%, compared with < 0.2% in HICs [26]. The diagnosis of intussusception is time-critical and hinges on ultrasound use. Ultrasound can also be used to guide hydrostatic or pneumatic reduction in the treatment of intussusception. Focusing on even this single condition, there is the potential to help improve diagnostic and referral pathways for children with intussusception in LMICs through more ubiquitous access to ultrasound skills for clinicians.

Given the lack of data regarding POCUS use in children’s surgery, we developed a global survey to evaluate existing practices, barriers to ultrasound access, training pathways, and the perceived utility of POCUS in our field.

## Methods

Following approval by the Global Initiative of Children’s Surgery (GICS) an electronic survey was disseminated via the GICS network and associated social media webpages in English language. Components of the survey were developed by pediatric surgeons with POCUS expertise. The term ‘POCUS’ was defined as the use of ultrasound by a (non-radiologist/non-sonographer) clinician in the assessment or treatment of a patient.

The survey assessed 4 areas:

- **Domain 1: Demographics**—information about the respondent’s role and country of practice.
- **Domain 2: Ultrasound Services**—information regarding ultrasound services, infrastructure, and barriers to access at the respondent’s institution.
- **Domain 3: POCUS Training** – respondent’s experience of POCUS training (or lack thereof) and utilization of POCUS skills in their current clinical practice.
- **Domain 4: POCUS Applications** – assessment of the respondent’s views on the clinical relevance of potential POCUS uses (‘applications’).

Twenty two potential POCUS applications were selected based on common pediatric conditions amenable to ultrasound diagnosis and the current use of POCUS in children’s healthcare as described in the literature. The survey was closed after 12 months. All responses were anonymous, and all respondents gave consent for publication of the survey findings.

There were 247 responses to the survey. These were collated in a secure electronic database. Of the 247 responders, 176 ([71.3%) were child surgeons; the remaining 71 worked in medical specialties. ‘Child surgeons’ were defined as those who identified as having specialized in ‘Pediatric Surgery’ or were from a different surgical specialty (i.e. general surgery, orthopedics, etc.) but routinely cared for children. Responses were compared between LMICs and HICs. Descriptive statistics were performed and chi-squared tests for statistical significance were used for categorical data. Statistical significance is indicated if p < 0.05 with a 95% confidence interval (CI).

## Results

### Domain 1—demographics

**Fig. 1:**
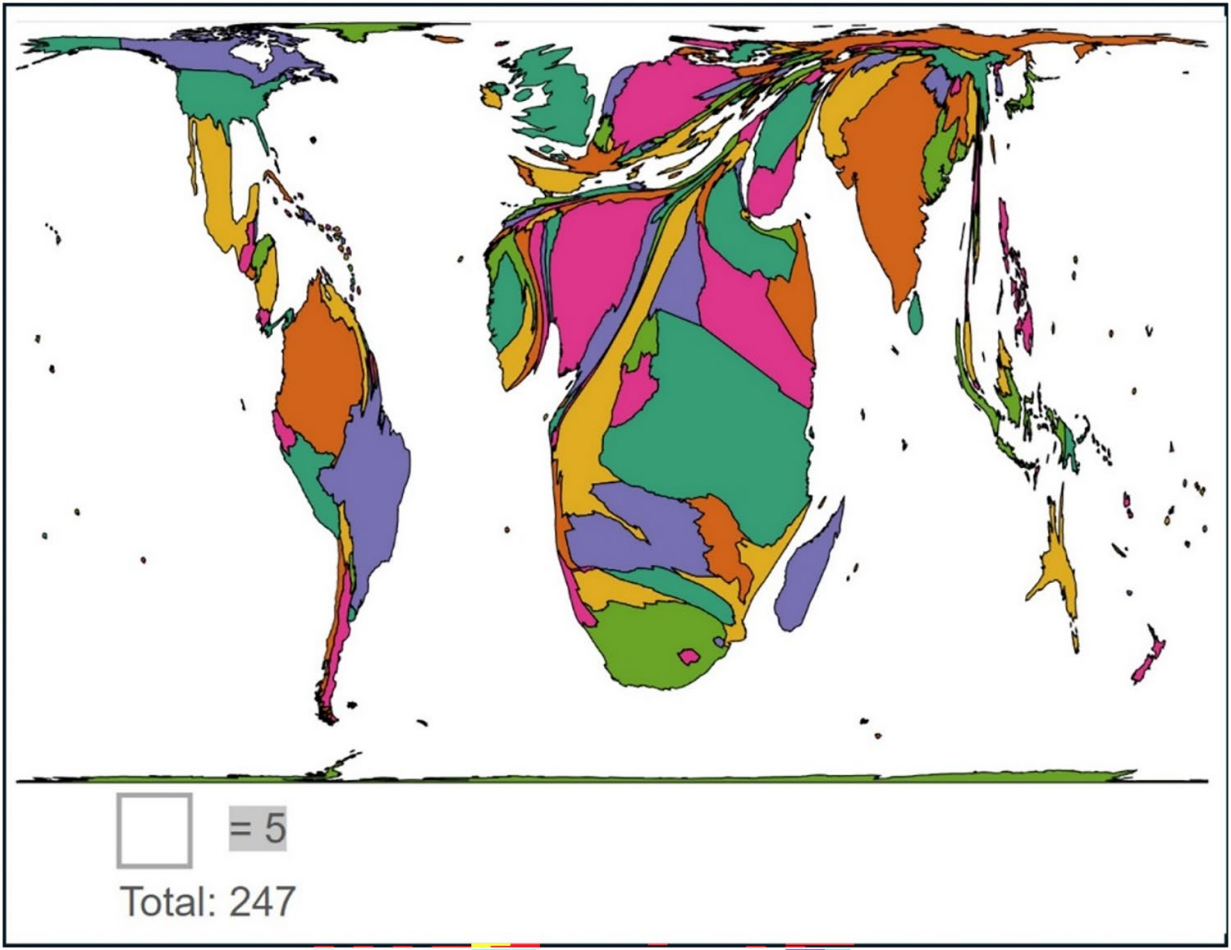
Cartogram showing the proportion of survey responses from different countries (Go-Cart program used to create cartogram)

**Fig. 2:**
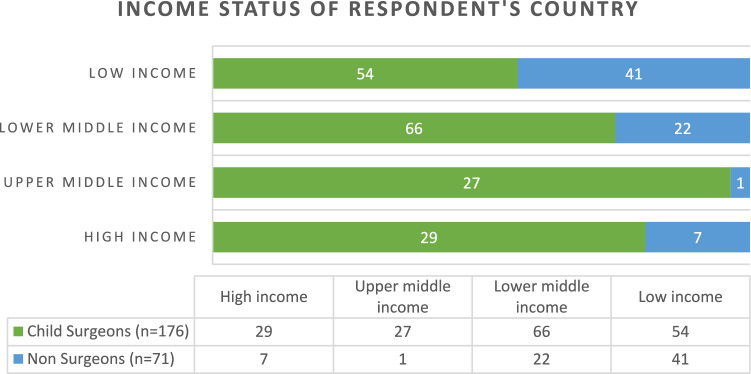
Number of responses according to the income level of the country as defined by the Work Bank

A total of 247 responses from 48 countries were received (additional data on a number of responses per country are given in Online Resource 1). These represented the continents of Africa, Asia, Europe, North and South America (Fig. 1). Countries were grouped by income level according to their official classification in the *World Bank List of Economies* [27] (Fig. 2). Most respondents were from LMICS 85.4% (211/247), with 14.6% (36/247) responses from HICs. Of the 176/247 child surgeons (*n* = 176), there was an almost equal split between consultant/attending level doctors 52.8% (93/176) and trainees/clinical officers 47.2% (83/176). Child surgeons predominantly worked in tertiary level institutions 83% (145/176); compared with secondary level 16% (28/176); and only 1% (3/176) from primary level healthcare facilities.

**Fig. 3:**
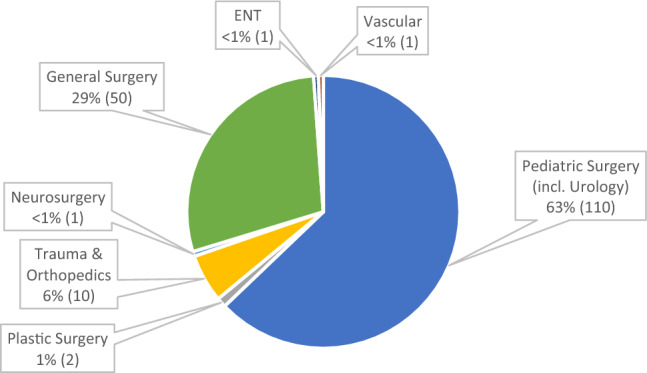
Breakdown of Child Surgeons specialties (*n* = 176)

Surgeons (*n* = 176) were mainly specialists in Pediatric Surgery 63% (110/176) or General Surgery 29% (30/176). The remaining responses were from 5 other surgical specialties (Fig. 3). All respondents routinely assessed or operated on children.

### Domain 2 – ultrasound services

We evaluated the availability of clinical investigations and ultrasound practices at the respondent’s hospital (Table 1). All had blood tests and ultrasound devices available at their institution (176/176). Cross-sectional imaging was available in all HIC settings (29/29) but only available in 65% (96/147) of LMICs. In terms of ‘normal’ ultrasound practices, in most respondent’s institutions ultrasound was typically performed by radiologists (or specialist sonographers) 64% (112/176). However, in almost a quarter of responses 24% (43/176), clinicians were the primary ultrasound operator, not radiologists. An equal mix of radiologists and clinician-led ultrasound was found in 11% (20/176). One respondent had access to ultrasound devices but no trained personnel to perform the skill. There was no statistically significant difference between LMICs and HICs in terms of who the principal ultrasound user was (P > 0.05; CI 95%).

**Table 1:** Ultrasound infrastructure and access at the respondent’s institution

	Total (*n* = 176)	HIC (*n* = 29)	LMIC (*n* = 147)
Available Investigations			
Blood tests	100% (176)	100% (29)	100% (147)
Radiographs	100% (176)	100% (29)	100% (147)
Ultrasound device/s	100% (176)	100% (29)	100% (147)
Cross-sectional imaging	71% (125)	100% (29)	65% (96)
Who normally performs ultrasound?			
Radiologist/sonographer only	64% (112)	48% (14)	67% (98)
Non-radiologist (doctor or clinical officer) only	24% (43)	7% (2)	12% (18)
Mix of both	11% (20)	45% (13)	20% (30)
No ultrasound users available	< 1% (1)	0% (0)	< 1% (1)
How often do you request an ultrasound?			
Almost daily	52% (91)	55% (16)	51% (75)
Multiple times per week	32% (56)	24% (7)	33% (49)
Multiple times per month	9% (15)	3% (1)	10% (14)
Rarely/never	7% (12)	7% (2)	4% (6)
N/A	< 1% (1)	7% (2)	1% (2)
No response	< 1% (1)	3% (1)	0% (0)
Time to ultrasound from request? *(Radiology Department)			
< 1 h	10% (17)	10% (3)	10% (14)
1–6 h	52% (92)	59% (17)	51% (75)
6–12 h	13% (23)	10% (3)	14% (20)
12–24 h	9% (16)	10% (3)	9% (13)
> 24 h	10% (17)	0% (0)	12% (17)
No guarantee it will be done	5% (8)	3% (1)	5% (7)
No radiology department	< 1% (1)	0% (0)	< 1% (1)
No response	1% (2)	7% (2)	0% (0)

In the clinical practice of child surgeons, ultrasound was a commonly used investigation, with 84% (147/176) requesting an ultrasound for a child either daily or multiple times per week—with no significant difference between LMIC and HIC groups (*p* > 0.05; CI 95%). In terms of ‘out of hours’ access to radiology-performed ultrasound, 19% (34/176) had no access, while 63% (111/176) could access these services but only in an emergency. We assessed the time for an emergency ultrasound from the request for an acute indication. In only 10% (17/176) of cases would emergency ultrasound be reliably performed < 1 h from request. For 40% (58/147) of LMIC respondents, an emergency ultrasound took more than 6 h to be performed; with 14% of these beyond 24 h from request (Table 1). No significant differences between LMIC and HIC were found across time intervals (*p* > 0.05).

**Fig. 4:**
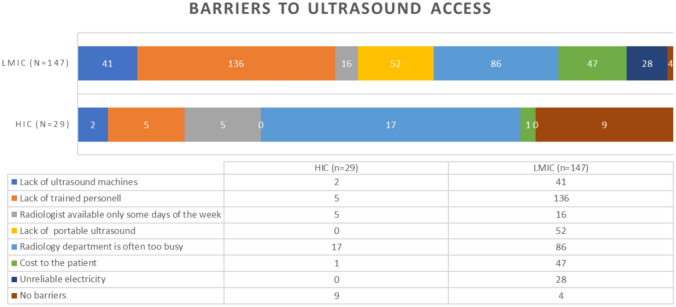
Barriers to ultrasound access described by respondent

We also investigated barriers to ultrasound access that child surgeons encountered at their hospital (Fig. 4). A lack of ultrasound-trained personnel and the workload of the radiology department were the commonest issues. In LMICs compared with HICs the following barriers were found to be statistically significant (CI 95%) – lack of machines (*p* = 0.0162); lack of trained personnel (*p* = 0.0001); lack of portable machines (*p* = 0.0001); cost to the patient (*p* = 0.0016); reliability of electricity source (*p* = 0.0016). HICS were more likely to have no barriers (*p* = 0.0001).

### Domain 3—ultrasound training

We asked respondents about their experience of ultrasound training, competence levels, and use of POCUS in their clinical management of children (Table 2). Approximately half of all respondents had received some kind of informal or formal ultrasound training (55%; 98/176). HIC surgeons were statistically more likely to be trained 24/29 (82.8%) compared with LMIC surgeons 74/147 (50.3%) (*p* = 0.001319; CI 95%). Of those who had received training (*n* = 98), most 46% (45/98) felt they had basic ultrasound competence; and almost half 47% (46/98) used POCUS daily or weekly in their practice. Self-perceived competence was associated with where the child surgeon used POCUS regularly in their clinical practice (*p* < 0.001; CI 95%). There were high levels of interest in attending a POCUS training course designed specifically for child surgeons, with 90% (159/176) saying they would be interested in attending such a course; 4% (7/176) had no interest; and 6% were unsure—no difference between LMIC and HIC groups (p > 0.05; CI 95%).

**Table 2:** Child surgeons (n = 176) experience of POCUS training, self-perceived competency, and clinical

POCUS Training	
Have you ever had any ultrasound training? (*n* = 176)	
No, I have never been trained	44% (78)
Yes, I attended a training course	27% (48)
Yes, I received informal training from colleagues	28% (50)
If you have received training, how competent do you feel using ultrasound to assess patients? (*n* = 98)	
I do not feel competent	32% (31)
I feel I have basic competence	46% (45)
I feel competent and can teach others	18% (18)
Non-response	4% (4)
If you have received training, how often do you personally perform ultrasound on your patients? (*n* = 98)	
Daily	26% (25)
At least once per week	21% (21)
At least once per month	24% (23)
Never	25% (24)
Non-response	5% (5)

### Domain 4—POCUS applications

**Fig. 5:**
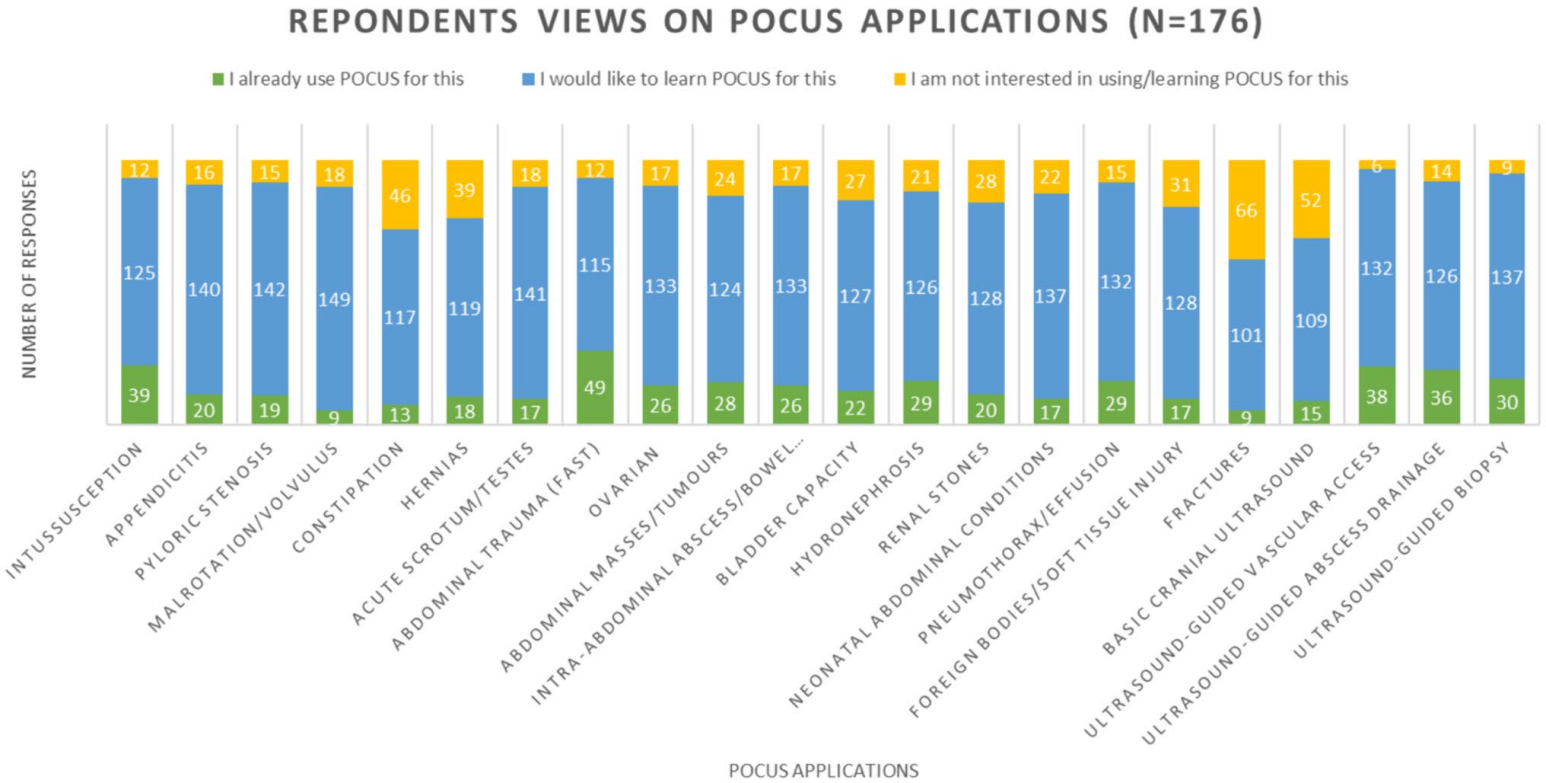
Response regarding applications of pocus, evaluating if respondents already used POCUS for this purpose or were interested or not interested in being trained to use POCUS for a particular condition/procedure

Respondents were asked to identify which of 22 potential POCUS applications they either already used POCUS for, would be interested in learning to use POCUS for, or had no interest in (Fig. 5). The top five most common uses of POCUS amongst child surgeons (*n* = 176) were FAST (focused assessment with sonography for trauma), intussusception, ultrasound-guided procedures, hydronephrosis, and pneumothorax detection. However, the most common applications respondents wanted to learn POCUS for were malrotation, pyloric stenosis, acute scrotum, appendicitis, and assessing the neonatal abdomen. The least useful applications included fractures, cranial ultrasound, constipation, hernias, and foreign bodies/soft tissue injury. Overall, there were high rates of interest in learning all of the 22 potential applications for POCUS, with a nadir of 57% (101/176) for fractures.

## Discussion

Within the pediatric global health community, there is increasing recognition of the magnitude of the burden of surgical disease particularly in LMICs where almost half the population are children [26, 28]. The Lancet Commission on Global Surgery 2015 has been instrumental in defining this problem and highlights issues such as lack of diagnostics as contributory factors, and imploring solutions that are interdisciplinary, innovative, and technology-enabled [28]. Additionally, one of the biggest challenges LMICs face in healthcare provision is the low ratio of doctors per capita compared with HICs [29]. To help address this, the global health community has endorsed ‘task shifting’ in many clinical areas [30–32]. Given the critical lack of pediatric radiologists in many low-income settings [20], there is a strong rationale for ‘task-shifting’ some of the most basic ultrasound applications to doctors and surgeons. This is the first study to attempt to delineate global practices and the views of child surgeons on the value of POCUS to children’s surgical care.

### Ultrasound use in child surgery

Our findings describe current norms and experiences of child surgeons concerning access to ultrasound services at their institution and the use of POCUS. We demonstrate that ultrasound in pediatric surgical care is critical in all settings, with child surgeons heavily reliant on this mode of imaging in their daily clinical practice (Table 1). Across both HIC and LMIC settings, the primary user of ultrasound (64%) was typically a radiologist or sonographer, as would be traditional. However, in almost a quarter of institutions surveyed, it was the norm for ultrasound to be clinician-led (POCUS) (Table 1). While the reason for this is unclear, it could reflect the general rise in POCUS use across the medical community in recent decades and an evolving status quo [33].

### Barriers

We also evaluated how easy it was for surgeons in their place of work to access traditional ultrasound services, with the majority (93%; 154/167) reporting some difficulty. The main barrier reported was not a lack of devices but rather a lack of ultrasound-skilled personnel (Fig. 4). Secondary to this was the workload pressure on local radiology departments (Fig. 4). Only 10% of respondents could reliably access emergency ultrasound within an hour from the time of request (Table 1). Our findings, therefore, demonstrate that the demand for ultrasound services in child surgery appears to outstrip the supply of trained ultrasound users. In LMIC institutions a significant additional barrier included the cost of ultrasound scans to families. In many institutions access to care requires payment ‘upfront’ which may render certain investigations inaccessible to poorer families. Equipping surgeons with POCUS skills to diagnose simple common pediatric conditions could help not only reduce the time to diagnosis and treatment of children but also improve the affordability of care.

### Cultural sensitivity

We asked respondents to describe (in free text) any other issues they experienced with POCUS use in their setting. A HIC respondent described that their radiology department was “unwilling for anyone else to do ultrasound”. In some regions (i.e. Europe/North America), such issues have been formally addressed through directives and guidance from Radiology associations that have published their endorsement of POCUS and encourage radiologists to support doctors who which to integrate it into their practice [34, 35]. A further consideration highlighted by our survey is region-specific cultural barriers to clinician-led ultrasound. A respondent working in India described that the regulation of ultrasound was stringent because of concerns over prenatal sex selection [36, 37]. In their setting, it was not advisable for surgeons outside of large training institutes to venture into ultrasound practice for this reason. It is important, therefore, that POCUS initiatives developed for surgeons must be contextually appropriate and sensitive to local culture. Ideally, a collaborative approach should be taken in developing POCUS programs, through proactive engagement with local radiology services and relevant governance bodies [9, 10, 34, 38, 39].

### Training

Our study indicates that most child surgeons (90%;159/176) would engage in formal POCUS training if a program with relevant content was made available. While a considerable proportion of surgeons already have some degree of ultrasound training, most have developed their skills informally. For those with training (formal or informal), the majority continued to practice their skills on a routine basis. However, we found that their perception of competence was associated with how frequently they practiced POCUS. Those who felt competent were more likely to use it regularly compared with those who did not. This finding is in keeping with published literature on POCUS training that emphasizes the importance of longitudinal training programs which over time build both competence and confidence in learners. This can be achieved through mentor-led learning and the use of simulation technology [40–42]. The latter, however, could be inaccessible to LMIC learners due to the large expense of current simulators. To better support LMIC POCUS learners there is a need to develop low-cost simulation technology.

### POCUS Applications

Our evaluation of clinical applications of POCUS found that surgeons who already practice POCUS most commonly use it for trauma (FAST), intussusception, and ultrasound-guided procedures. This is consistent with published reports of the most common protocolized uses of POCUS relevant to our specialty [43–46]. While respondents had high rates of interest in learning all 22 potential applications, the most valued were the diagnosis of malrotation/volvulus, pyloric stenosis, and acute scrotum. The accepted ‘gold standard’ for diagnosis of malrotation is fluoroscopy, with the role of ultrasound still under debate [47]. The interest shown in learning POCUS to diagnose malrotation (with or without volvulus) may represent a lack of access to fluoroscopy services in many respondents’ institutions. However, the evidence for the role of ultrasound is growing. The largest published meta-analysis of the diagnostic accuracy of ultrasound in malrotation/volvulus indicates the superiority of ultrasound which has a sensitivity of 94% (range 89% 97%; 95% CI) and specificity of 100% (range 97%-100%; 95% CI); compared with fluoroscopy sensitivity 91% (range 84%-96%; 95% CI) and specificity 94% (range 72% -99%; 95% CI) [47]. However, it does not account for the individual ultrasound user's accrued experience and expertise which could impact diagnostic accuracy. The interest in learning POCUS for pyloric stenosis and the acute scrotum may be due to the frequency with which pediatric surgeons manage these conditions, where diagnostic uncertainty may lead to delayed care or negative operative findings. A curriculum that aims to accredit child surgeons with POCUS competencies should aim to distinguish between ‘basic’ and ‘advanced’ applications so that they are feasible and safe to teach.

## Limitations

We acknowledge several limitations to this study. As with many surveys, there is likely to be a degree of selection bias given respondents, may be those who already have an interest in POCUS. The survey was circulated in the English language, which could have deterred non-English speakers from responding. However, of the 48 countries represented only 17 of these have English as an official language: with less than half 42% [103/247] of respondents working in an English-speaking country. Due to the low proportional response rate per country (range 1–17 responses per country), we cannot infer country-wide views on POCUS, our data instead likely reflects individual clinician views albeit within the area of child surgery.

## Conclusion

Our findings demonstrate the critical importance of ultrasound as a diagnostic tool in children’s surgery globally. Despite this, many child surgeons experience significant barriers to timely access to local ultrasound services for their patients. While approximately half of the respondents had some form of ultrasound training, the continued use of their skills was associated with self-perceived competence. Overall, we found high levels of interest amongst child surgeons in learning POCUS for relevant pediatric surgical applications. Training child surgeons in basic applications of POCUS could serve to improve access to diagnostics and reduce time to definitive care for children. Central to this intervention is linking this skill (POCUS) to the surgical care provider, thereby reinforcing the crucial link between the imaging and clinical findings. Further research is needed to evaluate the best training methods, mentorship, accreditation, and governance mechanisms for child surgeons learning POCUS.

## Supplementary Information

Below is the link to the electronic supplementary material.

Supplementary file1 (DOCX 20 KB)

## Data Availability

No datasets were generated or analysed during the current study.
