# Supplementary material for: Global survey on point-of-care ultrasound (pocus) use in child surgery
Source: Pediatr Surg Int. 2024 Sep 5;40(1):249. doi: 10.1007/s00383-024-05797-8 (PMC11377359; doi:10.1007/s00383-024-05797-8)
Supplement: Supplementary file 1 — Supplementary file1 (DOCX 20 KB) [file 383_2024_5797_MOESM1_ESM.docx]

**Supplementary Information (SI)**

**Title:** Global Survey on Point-of-Care Ultrasound (POCUS) use in Child Surgery

**Authors:** Gerlin Naidoo^1^, Mohammed Salim^2,3^, Andrew Jackson^2,3^, Ashok Handa^1^, Kokila Lakhoo^1,3^, Judith Lindert^4^

**Affiliations:**

^1^Nuffield Department of Surgical Sciences, University of Oxford, UK

^2^Paediatric Surgery Unit, Muhimbili National Hospital, Tanzania

^3^Muhimbili University of Health and Allied Sciences, Tanzania

^4^Department of Pediatric Surgery, University of Rostock, Germany

**Corresponding author contact details:**

Gerlin Naidoo (gerlin.naidoo@nds.ox.ac.uk"gerlin.naidoo@nds.ox.ac.uk; tel +447724712025)

Address: Nuffield Department of Surgical Sciences, University of Oxford, Room 6607, Level 6 John Radcliffe Hospital, Headington, Oxford, OX3 9DU, UK

**Online Resource 1** Responses per country

| **Country** | **LMIC/HIC** | **Child Surgeon Responses (n=176)** | **Total (Non-surgeons + surgeons) Responses (n=247)** |
| --- | --- | --- | --- |
| Austria | High income | 1 | 1 |
| Bangladesh | Lower middle income | 5 | 6 |
| Belgium | High income | 1 | 1 |
| Botswana | Upper middle income | 1 | 1 |
| Brazil | Upper middle income | 3 | 3 |
| Burundi | Low income | 17 | 25 |
| Cameroon | Lower middle income | 2 | 2 |
| Canada | High income | 1 | 1 |
| Chile | High income | 1 | 1 |
| Colombia | Upper middle income | 11 | 11 |
| DRC | Low income | 2 | 3 |
| Ethiopia | Low income | 3 | 3 |
| Germany | High income | 8 | 13 |
| Ghana | Lower middle income | 1 | 3 |
| Guatemala | Upper middle income | 1 | 1 |
| India | Lower middle income | 8 | 8 |
| Italy | High income | 1 | 1 |
| Kenya | Lower middle income | 6 | 7 |
| Liberia | Low income | 2 | 3 |
| Lithuania | High income | 1 | 1 |
| Madagascar | Low income | 0 | 3 |
| Malawi | Low income | 10 | 21 |
| Malaysia | Upper middle income | 1 | 1 |
| Malta | High income | 1 | 1 |
| Mexico | Upper middle income | 2 | 2 |
| Netherlands | High income | 1 | 1 |
| Nepal | Lower middle income | 0 | 1 |
| Nicaragua | Lower middle income | 1 | 3 |
| Nigeria | Lower middle income | 13 | 13 |
| Pakistan | Lower middle income | 3 | 3 |
| Peru | Upper middle income | 0 | 1 |
| Philippines | Lower middle income | 1 | 1 |
| Rwanda | Low income | 1 | 3 |
| Saudi Arabia | High income | 1 | 1 |
| Sierra Leone | Low income | 16 | 26 |
| Singapore | High income | 1 | 1 |
| Somalia | Low income | 1 | 3 |
| South Africa | Upper middle income | 8 | 8 |
| Spain | High income | 1 | 1 |
| Sri Lanka | Lower middle income | 1 | 1 |
| Tanzania | Lower middle income | 14 | 29 |
| Uganda | Low income | 1 | 3 |
| UK | High income | 9 | 11 |
| USA | High income | 1 | 1 |
| Vanuatu | Lower middle income | 1 | 1 |
| Yemen | Low income | 1 | 2 |
| Zambia | Lower middle income | 8 | 8 |
| Zimbabwe | Lower middle income | 2 | 2 |
